# Identifying Patients with Heart Failure Eligible for Guideline-Directed Medical Therapy

**DOI:** 10.1089/pop.2024.0132

**Published:** 2024-12-04

**Authors:** Samantha Subramaniam, Shahzad Hassan, Ozan Unlu, Sanjay Kumar, David Zelle, John W. Ostrominski, Hunter Nichols, Jacqueline Chasse, Marian McPartlin, Megan Twining, Emma Collins, Echo Fridley, Christian Figueroa, Ryan Ruggiero, Matthew Varugheese, Michael Oates, Christopher P. Cannon, Akshay S. Desai, Samuel Aronson, Alexander J. Blood, Benjamin Scirica, Kavishwar B. Wagholikar

**Affiliations:** 1 Accelerator for Clinical Transformation, Brigham and Women’s Hospital, Boston, Massachusetts, USA.; 2 Division of Cardiovascular Medicine, Brigham and Women’s Hospital, Boston, Massachusetts, USA.; 3 Division of Endocrinology, Diabetes and Hypertension, Brigham and Women’s Hospital, Boston, Massachusetts, USA.; 4 Division of Pharmacy, Brigham and Women’s Hospital, Boston, Massachusetts, USA; 5 Laboratory of Computer Science, Massachusetts General Hospital, Boston, Massachusetts, USA; 6 Harvard Medical School, Boston, Massachusetts, USA.

**Keywords:** electronic health records, population health management, guideline adherence, health information systems

## Abstract

A majority of patients with heart failure (HF) do not receive adequate medical therapy as recommended by clinical guidelines. One major obstacle encountered by population health management (PHM) programs to improve medication usage is the substantial burden placed on clinical staff who must manually sift through electronic health records (EHRs) to ascertain patients’ eligibility for the guidelines. As a potential solution, the study team developed a rule-based system (RBS) that automatically parses the EHR for identifying patients with HF who may be eligible for guideline-directed therapy. The RBS was deployed to streamline a PHM program at Brigham and Women’s Hospital wherein the RBS was executed every other month to identify potentially eligible patients for further screening by the program staff. The study team evaluated the performance of the system and performed an error analysis to identify areas for improving the system. Of approximately 161,000 patients who have an echocardiogram in the health system, each execution of the RBS typically identified around 4200 patients. A total 5460 patients were manually screened, of which 1754 were found to be truly eligible with an accuracy of 32.1%. An analysis of the false-positive cases showed that over 38% of the false positives were due to incorrect determination of symptomatic HF and medication history of the patients. The system’s performance can be potentially improved by integrating information from clinical notes. The RBS provided a systematic way to narrow down the patient population to a subset that is enriched for eligible patients. However, there is a need to further optimize the system by integrating processing of clinical notes. This study highlights the practical challenges of implementing automated tools to facilitate guideline-directed care.

## Introduction

Heart failure (HF) remains a public health problem of major proportion, and the national burden is estimated to increase by 25% by 2030.^1,2^ Although many new therapies for managing patients with HF have shown benefit and are accordingly recommended by current practice guidelines, guideline-directed medical therapy (GDMT) remains significantly underutilized in clinical practice.^3^ A key challenge in implementing clinical interventions to improve GDMT is that clinical staff are required to exert extensive manual effort to review electronic health records (EHR) to determine a patient’s guideline eligibility. To address this issue, we have developed a rule-based system (RBS) for searching the EHR to find patients who are eligible for the HF guidelines.

## Background and Significance

More than 25 years after its designation as an emerging epidemic,^4^ HF remains a clinical and public health problem of major proportion. Approximately 6.5 million adults in the United States currently have HF, and nearly 0.9 million new cases are diagnosed annually.^1,5^ The lifetime risk of developing HF at age 40 is 20%.^6^ Projections show that the prevalence of HF will increase by 46% from 2012 to 2030, resulting in >8 million adults with HF.^7^ Until 2012, the hospitalizations for HF were decreasing; however, the trend has reversed, largely due to the aging population. The total cost of HF care in the United States exceeds $30 billion annually, including health care services, medications, and missed days at work.^1,8^

Despite systematic efforts by the medical community over the last 2 decades, progress in reducing the incidence and improving the outcomes of HF has not been commensurate with the resources dedicated to its management. Current medical therapies have shown benefits across the spectrum of HF phenotypes based on the ejection fraction (EF): Heart failure with reduced ejection fraction (HFrEF), heart failure with mildly reduced ejection fraction (HFmrEF), and heart failure with preserved ejection fraction (HFpEF). Multiple professional societies recommend medical therapies with proven benefits for patients with HF to reduce hospitalizations, prolong survival, and improve quality of life.^10^ For patients with HFrEF, quadruple therapy consists of (1) renin–angiotensin system inhibition with angiotensin receptor–neprilysin inhibitors (ARNI), angiotensin-converting enzyme inhibitors (ACEi), or angiotensin II receptors blockers alone; (2) beta-blockers; (3) mineralocorticoid receptor antagonists (MRAs); and (4) the sodium–glucose co-transport inhibitors (SGLT2i), all receiving Class 1 recommendations with Level of Evidence A.^9,11^ For HFmrEF, SGLT2i are given Class 2a recommendations, while ARNI, ACEi, ARB, MRA, and beta-blockers receive Class 2b recommendations. For HFpEF, SGLT2i are recommended (Class 2a) with MRA and ARNIs receiving Class 2b recommendations.^9^ The rapid initiation and titration of GDMT are associated with improved outcomes.^12^ Despite robust evidence of clinical benefits and guidelines from professional societies, GDMT remains significantly underutilized in clinical practice.^13,14^

Efforts to improve GDMT in patients with HF are ubiquitous across health care systems. The interventions include clinician education and audits, nurse-led follow-ups after discharge,^15^ protocolized visits with cardiologists after hospital discharge,^16^ EHR-embedded alerts,^17,18^ patient registries, and multidisciplinary team-based programs.^19^ Protocolized visits with cardiologists and interventions based on multidisciplinary teams have been found to have a better impact than clinician alerts or quality initiatives that focus on education and audit-feedback processes alone.^16^ The effort presented in this article has been carried out to facilitate a population health management (PHM) program at Brigham and Women’s Hospital (BWH), called the Cooperative Program for ImpLementation of Optimal Therapy in Heart Failure.^20,21^ It is a pragmatic, randomized implementation trial (NCT05734690),^24^ in which non-clinician staff (navigators), who are especially trained to approach patients, communicate with patients via phone and titrate the patients’ medications working alongside pharmacists and under the supervision of an advanced practice provider and a cardiologist.^22,23^ Navigators interact with participants via phone to implement medication changes and monitor adverse events, blood pressure, and lab results. Upon patient acceptance, navigators inform pharmacists who then sent the new prescription.

A key obstacle to the PHM program like other hospital-based initiatives to improve GDMT is the effort of reviewing charts to identify the patients who are on suboptimal GDMT. To facilitate the implementation of the PHM, the study team developed and deployed an RBS for finding patients with HF who may be potentially eligible for receiving medications as recommended by clinical guidelines. This article describes the methodology to create the RBS and presents an evaluation of the RBS after 1 year of deployment. Findings of this study will inform the development of similar systems to facilitate PHM programs at other institutions.

## Methods

**FIG. 1.**
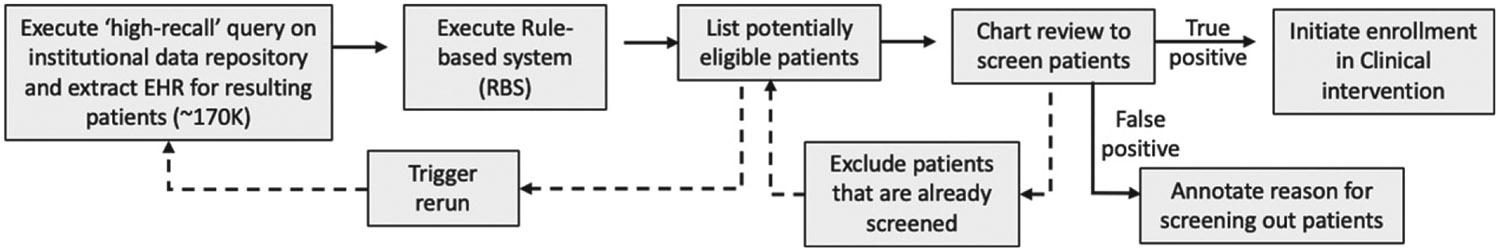
Deployment of the rule-based system to find patients for enrollment into the remote medication management program.

The study team developed an RBS to identify patients who were not receiving guideline-directed medications, to facilitate these patients’ enrollment into the PHM program at BWH. The RBS processed EHR from the institution’s enterprise data warehouse, to create the list of potentially eligible patients. The list was made available to the navigators who reviewed each patient’s chart to determine the patient’s true eligibility. This study was approved by the institutional review board of BWH (Figure 1). The following sections describe the methodology for developing and evaluating the RBS.

### Steps to develop the rule base

1. **Identify criteria in the HF medication guidelines.** The study team reviewed the American Heart Association and American College of Cardiology guidelines for the criteria to identify patients eligible for GDMT. The guidelines classify symptomatic patients with HF into 2 groups by EF and excluded patients with contraindications to GDMT or a higher risk of adverse medication reactions. In addition to the guidelines, the team developed criteria to facilitate the PHM program’s logistics. For example, the program required the consent of principal cardiology care provider for each of the eligible patients. Accordingly, the cohort was restricted to patients whose cardiology care provider (cardiologist or primary care provider) was within the Mass General Brigham health system.
2. **Decompose criteria into granular components (or variables) that can be derived from the EHR.** Next, we decomposed the criteria into granular components. For instance, the EHRs do not contain an explicit variable corresponding to the criteria of “symptomatic HF.” Hence, this criterion was decomposed into a combination of mention of HF in the problem list and prescription of “loop diuretic” medication in the last 2 years. Together these can serve as a surrogate to determine symptomatic HF.
3. **Value sets**. EHR is generally stored using a granular format. For instance, the fact that a particular patient is prescribed a beta-blocker is recorded as a tuple of the patient’s medical record number, prescription date, and brand and dose of the medication. The brand and dose of the medication are catalogued using an alphanumeric code system such as RXNORM or National Drug Code. Hence, to abstract this information into coarser concepts, the medication codes need to be mapped to coarse concepts like medication class. For instance, the codes for the brand name “Toprol 25 mg” are mapped to “metoprolol succinate 25 mg,” which is further grouped into the beta-blocker medication class. Value sets were developed for diagnoses, laboratory values, and medication classes of interest. The value sets used in the RBS are included in Supplementary Data S1.
4. **EHR data extracts.** The study team reviewed the EHR data elements to identify extracts/sections that can be used to determine the variables defined in Step 2. Multiple sections of the EHR can be useful to determine a particular variable. For instance, the variable to denote that a patient has amyloidosis can be determined by the presence of the billing code for amyloidosis in a hospital encounter or explicit documentation of amyloidosis by the care provider in the problem list or progress notes. The variables were mapped into one or more sections of the health record.
5. **Exclude patients receiving adequate medications.** Finally, the study team implemented the guideline logic to filter out the subset of patients who were already receiving GDMT. They excluded patients who were currently on medications from all the classes of GDMT. However, the analysis did not consider whether the dosage of the medications was as recommended by the clinical guidelines.
6. **Implement rule base using SQL and Python.** The logic rules developed in the above steps were implemented in SQL and Python. The resulting rule base comprises 5 components: (1) retrieve EHR, (2) apply value sets to extract data, (3) derive components/variables for guideline criteria, (4) apply criteria to determine eligibility, and (5) exclude patients already receiving GDMT.

### Deployment and evaluation of RBS

The study team evaluated the RBS’s performance by deploying it in a real-world setting of the PHM program at BWH. They created a high-recall query to find the medical record numbers of patients who had a transthoracic echocardiogram in the health system and met the criteria of age between 18 and 90 years, alive, and English- or Spanish-speaking. This query was executed on the institutional research data repository to obtain the “denominator set” of patients for this study. The EHR data for all patients in the denominator population were extracted, and then the RBS was executed on the extracted data to create a list of patients potentially eligible for GDMT.

The EHR data extract for patients was refreshed approximately once every 2 months, and the RBS was rerun to obtain a new list for manual screening. The navigators used an electronic checklist while performing the chart review to annotate the presence or absence of a program criterion. The checklist ensured a consistent approach to record the results of the manual chart review.

We measured the RBS’s performance as the percentage of patients determined to be truly eligible by manual chart review performed by the program navigators. Furthermore, we examined the navigator’s annotations for the false-positive patients, to identify ways to further improve the RBS.

## Results

**FIG. 2.**
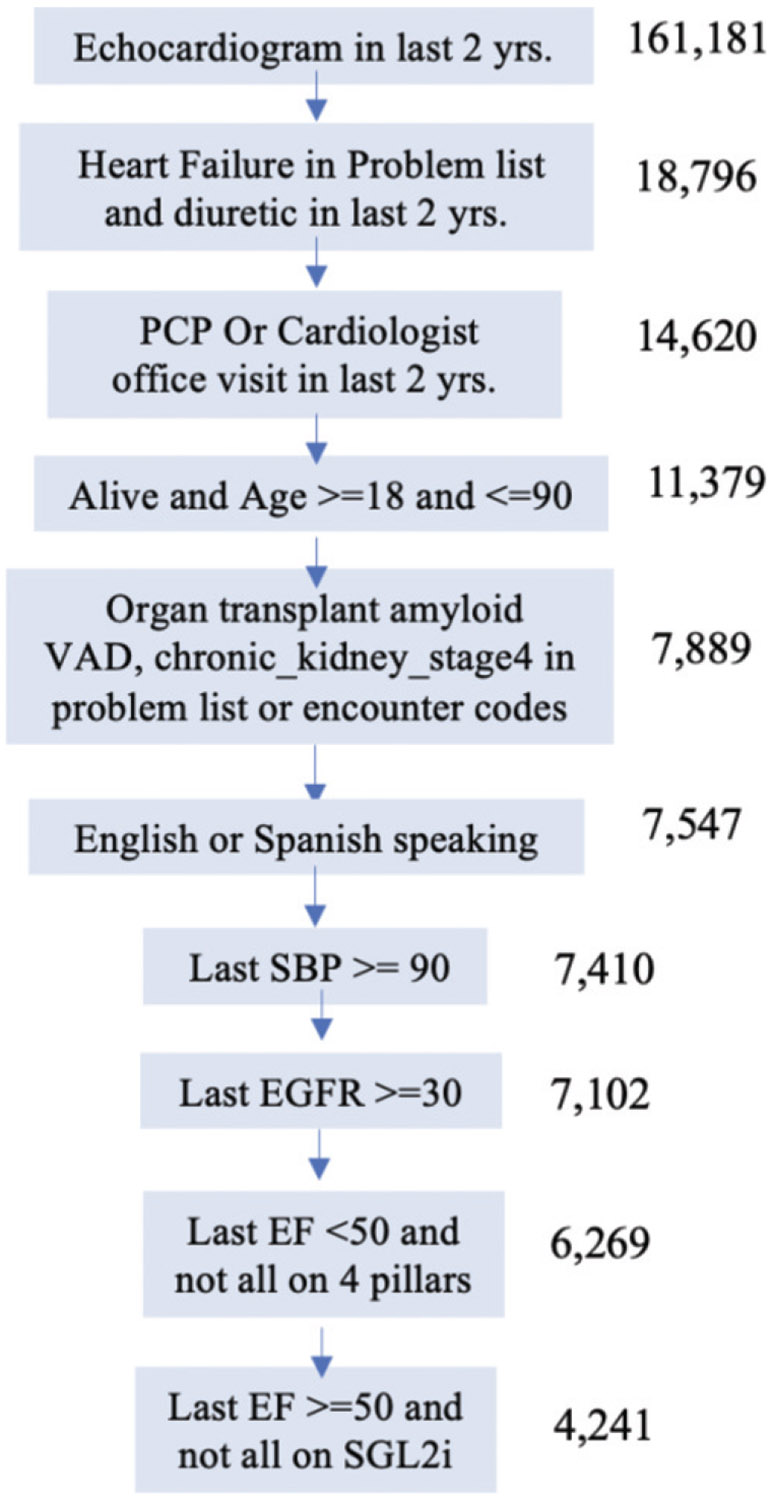
Consort diagram showing the patient counts during a typical execution of the RBS. RBS, rule-based system.

**FIG. 3.**
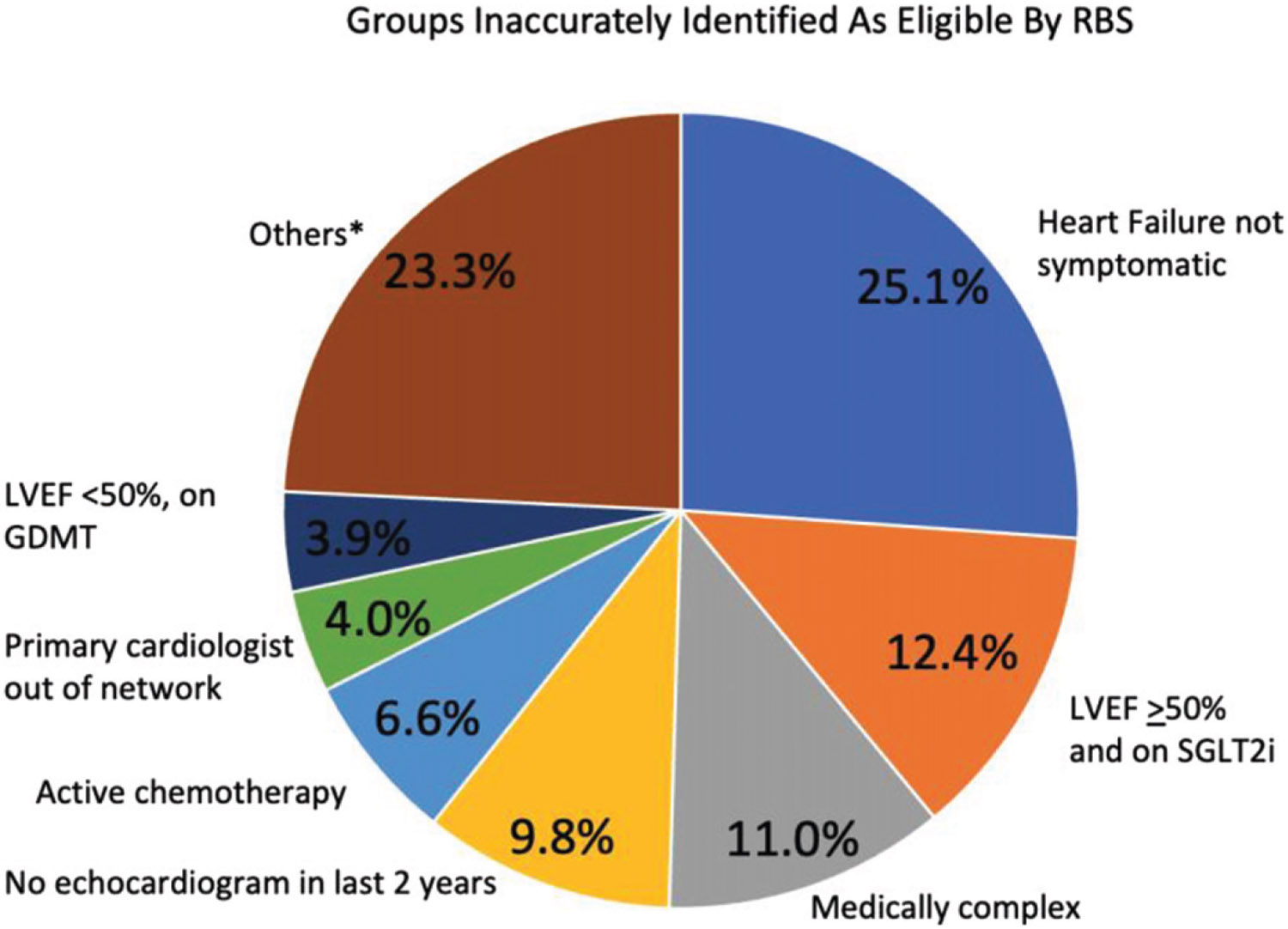
Summary of reasons for false positives from RBS determined by manual chart review. Over 25% of patients identified as eligible by RBS were found to not have symptomatic HF, the criteria of which included clinical symptoms documented in clinician notes, use of diuretics, or acute HF hospitalization within last 2 years from time of screening. RBS did not process clinical notes to identify patients with symptomatic HF. HF, heart failure; RBS, rule-based system.

Each execution of the high-recall query resulted in a denominator set of approximately 161,000 patients, from which the RBS would short-list approximately 4200 (2.6%) patients for screening (see Fig. 2). In the period from January 1, 2023, to December 31, 2023, the navigators reviewed the electronic charts of 5660 patients identified by the RBS. They “screened-in” 1754 patients (1754/5460 = 32.1%), and the remaining 3706 patients were deemed “screened-out.” Navigators indicated the reasons for screening out 3177 patients (Table 1, Table 2 and Fig. 3).

Although there can be multiple reasons for screening out a patient, the navigators generally terminated their chart review when they encountered a reason for screening out, so usually only 1 screen-out reason was indicated per patient. The distribution of the number of screen-out reasons is shown in Table 3.

## Discussion

We developed an RBS that automatically processed EHR to identify a cohort of patients with HF who were not receiving GDMT. The system was deployed in a real-world setting to find patients for enrollment into a PHM program at BWH.

Development of the RBS was necessary given the complexity of the guideline logic for determining the optimal medications based on the EHR data. Breaking up the logic into individual queries for execution on the EHR involves considerable manual effort and introduces a risk of errors.^25^ Rule-based systems have been used to implement clinical guidelines in infectious disease,^26,27^ prescription,^28^ and cancer surveillance.^29-31^ The approach of combining administrative codes and search terms to model clinical guidelines has been utilized effectively in previous studies.^32^

Furthermore, the institutional enterprise data warehouse (EDW) has limited computational power to execute the multitude of steps in the guideline logic. Given the practical limitations on performing the computations in the EDW, we implemented the RBS externally to the EDW. We executed the high-recall query on the EDW to create a denominator cohort and retrieved this cohort’s data onto an institutional server having sufficient computational resources to perform the computations.

The RBS effectively narrowed down the denominator cohort of approximately 161,000 patients to a smaller list of approximately 4200 (2.6%) patients to facilitate the PHM program. The RBS was evaluated to have an accuracy of 32.1%, which helped operationalize the PHM program. Without the RBS, the program team would have to use the conventional approach of querying the EDW to narrow down the denominator population to a smaller set, which would have been cumbersome given the complexity of the criteria and limits on computational processing in the EDW. Furthermore, it would be practically impossible to sift through all the patients’ charts. Alternatively, the program team would have to rely on referrals from the clinicians or the deployment of clinical alerts in the EHR, which would lead to an additional burden on the clinicians potentially contributing to their “alert fatigue” and burnout. Hence, the RBS provides a systematic way to narrow down the cohort to a subset that is enriched for eligible patients, as compared with conventional approaches.

The approach of representing the guideline logic as rules and breaking down the logic systematically into steps that are assembled to determine individual criteria provides a mechanism to increase the granularity of the analysis. This contrasts with the low-complexity queries that are typically possible with conventional queries of an EHR or data warehouse. The rule-based approach provides a robust mechanism to validate rule output at a detailed level and fix errors in the system implementation. Additionally, it allows the computation to be staged in order to apply the logic efficiently.

There have been substantial efforts in the last several decades to implement the logic recommended by clinical guidelines.^33,34^ Several implementations have been developed to facilitate clinical decision support, measure care quality, and create cohorts for research, leading to an evolution of frame-works and standards such as Fast Healthcare Interoperability Resources (FHIR) and Clinical Quality Language (CQL).^35-37^ However, the infrequency of reports on real-world evaluations of these system using these standards indicates that these technologies are still not mature, and a stronger evidence base is needed to foster the use of standards in Clinical Decision Support design and implementation.^38^ Given the steep learning curve to implement systems using the proposed standards and given the standards’ relative immaturity, we currently did not invest effort in standardizing our RBS to FHIR and CQL standards. However, we believe that this step is essential to facilitate dissemination.

A high proportion of the false positives (39%) was due to incorrect detection of symptomatic HF (25%) and medications (17%). The criterion of symptomatic HF is a requirement in the guidelines for initiating medications, as there is a lack of consensus that asymptomatic patients with reduced Left ventricular ejection fraction (LVEF) can benefit from medications. However, it is challenging to determine this criterion from EHR data. Hence, the prescription of diuretics is used as a surrogate, as they are used to mitigate the symptoms of HF that usually occur due to fluid overload. Symptomatic HF is challenging to detect, as it is a clinical diagnosis that is not explicitly documented in the EHR. It is diagnosed with symptoms of shortness of breath, dyspnea on exertion, orthopnea, paroxysmal nocturnal dyspnea, lower extremity edema, and symptoms of fluid overload including weight gain in a short time, imaging pulmonary edema, pleural effusion, rales crackles, and pitting edema. The symptoms are typically described in clinical notes that are currently not processed in the RBS, which is the likely reason for the high proportion of false positives.

Similarly, the difficulty of accurately extracting the medication history may possibly be due to patients receiving prescriptions at another hospital. This information for prescriptions from external sites is not available in the instructional research database but is likely documented in the clinical notes, which were not processed in the RBS.^39^ The authors anticipate that the false positives attributed to symptomatic HF and medication history can be improved by integrating the RBS with conventional natural language processing techniques as well as the use of large language models.^40-43^

The RBS included several criteria necessary to facilitate the medication management intervention. For instance, the criterion for restricting the program to patients who knew English or Spanish was due to the IRB’s consideration that language interpreters are essential for effective communication with the patients. Furthermore, the criterion to exclude patients receiving the cardiology care from outside the hospital was to simplify obtaining the provider’s assent to enroll their patient into the medication management program. The criterion of basic metabolic panel in the last year is to ensure that the baseline parameters of the patient are available before initiating new medications for the patient.

The exclusion criteria in the RBS were designed to exclude patients who had complex decision scenarios that require specialist expertise. This entailed excluding patients with cardio-myopathy, amyloid or congenital heart disease, transplant, last systolic blood pressure <90, ventricular assistive device, advanced kidney disease (estimated glomerular filtration rate [EGFR] <40 or active dialysis), or active chemotherapy. The evaluation demonstrated that this group of patients was effectively excluded as it accounted for only a total of 14% of the false-positive cases.

This study did not include rare exclusion criteria in the RBS, such as pulmonary arterial hypertension, end-of-life care, more than 4 hospitalizations in the last 6 months, and type 1 diabetes, which together constituted 6% of the false positives. The low prevalence supports our approach of bypassing the programming effort to implement these criteria in the RBS.

The value sets are provided in Supplementary Data S1 to facilitate the implementation of similar systems at other institutions. These value sets need to be mapped to the data elements in the local EHR repository to implement the RBS.

### Limitations

A limitation of our study is that it only considered whether the patients are on a particular class of medication for determining if they are receiving appropriate GDMT and did not consider the recommended dosage for the medication. Hence, there are potentially more patients who could benefit by up-titration of their medication dosage than the patients identified by our RBS.

Another limitation is that the study only considered structured variables from the EHR for executing the RBS. As indicated in our analysis, inclusion of data from clinical notes can potentially improve the quality of the variables in the RBS and improve the accuracy of the RBS. In particular, there is potentially more information in clinical notes for determination of symptomatic HF and medication history.^40^

## Conclusion

The RBS provided an effective mechanism to model and execute the guideline logic in comparison with the conventional approach of querying the institutional health record repository. It provided a systematic way to narrow down the patient population to a subset that is enriched for eligible patients. However, there is a need to further optimize the system by integrating processing of clinical notes to improve the screening rate and to enhance the efficiency and impact of the clinical intervention. Overall, our study highlights the practical challenges of implementing automated tools to facilitate guideline-directed care.

The definitions of the value sets for deriving the variables in the rule base are provided in Supplementary Data S1, along with a query for the guideline criteria (https://github.com/dschc/supplementary_material/blob/main/supplementary.zip).

## Supplementary Material

supplementary

Supplementary Data S1

## Figures and Tables

**Table 1.** Demographics of Screened Cohort

Parameter	Counts	Percent (%)
Marital status		
Married	2810	51.5
Single	1191	21.8%
Divorced	521	9.5%
Others	938	17.2
Language		
English	5260	96.3
Spanish	132	2.4
Others	248	4.5
Ethnicity		
Hispanic	284	5.2
Non-Hispanic	4972	91.1
Others	204	3.7
Race		
Caucasian	4616	84.5
African	438	8.0
Others	406	7.4
Sex		
Female	2629	48.2
Male	2831	51.8
Total	5460	100

**Table 2.** Distribution of Reasons for Screening Out False-Positive Cases

Reason	Exclusion	Count	Percent
Not symptomatic for HF	N	894	25.1
LVEF > 50 and on SGLT2i	Y	442	12.4
Complicated scenario	Y	392	11.0
No echocardiogram in last 2 years	N	349	9.8
Active chemotherapy	Y	236	6.6
MGH cardiologist	Y	142	4.0
LVEF < 50 and on GDMT or intolerant	Y	138	3.9
Not receiving cardiology care at institution	N	133	3.7
No basic metabolic panel in 1 year	N	132	3.7
Aortic stenosis	Y	114	3.2
History of cardiomyopathy	Y	88	2.5
Transplant	Y	61	1.7
Pulmonary arterial hypertension	Y	58	1.6
Age outside range (18–90 years)	N	56	1.6
End-of-life care	Y	51	1.4
EGFR < 30	Y	49	1.4
Last systolic BP < 90	Y	42	1.2
Hospitalized 4 or more times in last 6 months	Y	42	1.2
Amyloid heart disease	Y	29	0.8
Ventricular assistive device	Y	28	0.8
Not English- or Spanish-speaking	N	25	0.7
Type 1 diabetes	Y	23	0.6
Congenital heart disease	Y	15	0.4
Active dialysis	Y	7	0.2
Pregnant or breastfeeding	Y	7	0.2
Intravenous inotrope	Y	3	0.1

BP, blood pressure; GDMT, guideline-directed medical therapy; HF, heart failure; SGLT2i, sodium–glucose co-transport inhibitors; LVEF, left ventricular ejection fraction; EGFR, estimated glomerular filtration rate; MGH, Massachusetts General Hospital.

**Table 3.** Distribution of Number of Screen-out Reasons Annotated by the Clinical Staff During the Manual Chart Review

Reasons	Counts
1	2843
2	295
3	35
4	3
6	1
Total	3177
